# Introducing a Synchronous Workshop Feedback Model for OSCE Development in an International Education Partnership

**DOI:** 10.1007/s40670-024-02136-3

**Published:** 2024-08-29

**Authors:** Anjali Gondhalekar, Zakia Arfeen, Lamiaa Mohsen, Marwa el Shabrawy, Hend Mokhtar, Mohammed Ahmed Rashid

**Affiliations:** 1 UCL Faculty of Medical Sciences, University College London, London, UK; 2 School of Medicine, New Giza University, Cairo, Egypt

**Keywords:** Feedback, OSCE, Partnership

## Abstract

University College London (UCL) and Newgiza University (NGU) have been in an academic collaboration since 2016. We describe the introduction of a real-time feedback model for OSCE assessments within this partnership. We developed a workshop for faculty members at UCL and NGU to co-develop OSCE stations for use in final year summative exams at NGU. Structured discussions in small groups about content and logistics of each proposed station enabled the teams to jointly finalise the assessment blueprint and station list. A synchronous workshop model has since become a popular method to improve assessment quality and co-develop assessments in international settings.

## Background

Objective structured clinical examinations (OSCEs) are widely used in Health Professions’ Education globally as a tool to test specific skills relevant to clinical practice. Whilst in some countries they have been used for decades, in others they have been introduced relatively recently. In the UK for example, the implementation of integrated OSCE stations where multiple tasks are combined in a single practical station is commonplace [1]. In comparison, for many educational programmes and institutions elsewhere in the world, this integrated OSCE style is still novel and faculty members are unlikely to be familiar with the operational approach to this style compared to the more traditional long case or viva styles of practical assessment [2].

International partnerships in medical education are an increasingly popular tool to enable new and existing organisations to raise standards [3] with assessment being a particularly important area in these partnerships. The UCL Medical School’s Centre for International Medical Education Collaborations (CIMEC) works collaboratively with institutions around the world to develop tailored medical educational programmes by co-developing and embedding high-quality education and assessments [4]. Collaborating medical institutions are located in various countries and are often at the early stages of developing and establishing a medical educational curriculum and assessment structure. Thus, they often value the knowledge and expertise that the UCL Medical School CIMEC team can provide. All collaborations aim to co-create high-quality, sustainable and locally customised education programmes [4]. Assistance with the development of assessments has encompassed various areas of the partnership both in assessment design and delivery of high-quality written and practical assessment items including OSCEs, where feedback and experiential knowledge are often highly valued by collaborators [5].

Over the past 7 years, Newgiza University (NGU) and UCL have worked in an international academic collaboration in the development and delivery of its undergraduate medicine, dental and pharmacy programmes. During this time, senior faculty and staff from UCL have worked with NGU, to strategically plan and organise the new programmes by setting up appropriate infrastructure, developing curricula and resources and ensuring education quality assurance. This enduring partnership has been strengthened by collaboration between faculties [6]. Providing feedback is a crucial step in the development of an OSCE station for quality assurance and the peer-review process can be helpful in this [7]. Together with NGU, UCL have reflected on processes and challenged their existing models of assessment feedback.

Faculty development has been shown to increase OSCE item quality [8] and the collaboration between UCL and NGU has always worked towards strong quality assurance measures. Historically, when developing OSCE assessment material with NGU, station authors from NGU would draft and submit OSCE stations to the UCL team based on previously delivered faculty development sessions. The UCL academic team would subsequently provide tailored review, feedback and quality assurance for each station before it was returned electronically to the faculty members to make amendments and finalise the draft for delivery in the examinations. Feedback would be taken at the discretion of the NGU station authors and leadership teams and implemented according to their needs, with little further dialogue between author and reviewer.

NGU and UCL worked collaboratively to identify some of the key challenges posed by this methodology. Firstly, the feedback was not always timely [9] as the method of writing OSCE stations; their dissemination for review and duration for turn around of feedback can be time consuming. Moreover, feedback provided was unidirectional in delivery, which we know is certainly a historically conceptualised process in medical education [1], and maybe perceived as less effective in the spirit of collaboration. This approach has also posed difficulties for the UCL team to confirm whether the feedback has been fully understood or can be readily applied to enable the changes to be implemented accurately. This lack of a two-way dialogue in provision of feedback also failed to provide NGU with an opportunity to voice any adaptations that they required to follow through with advice on an OSCE station to make it relevant to the local community. For example, there may be differences in the availability of certain resources such as specific prosthetic models or access to simulated patients with relevant training to deliver the OSCE station.

As mentioned, the use of integrated OSCE stations remains a novel approach to assessment for some nations, but global partnerships such as that between NGU and UCL work to continually collaborate and produce high-quality assessment items. This difference in the traditional model of writing OSCE stations has heightened the need for novel approaches to improve the feedback provided and greater two-way dialogue between UCL and NGU.

## Activity

To improve upon this, a synchronous workshop was co-designed by UCL and NGU teams, which adopted a new teaching and learning approach of providing real-time feedback on assessment materials as a second ‘layer’ of feedback especially for the high stakes’ final year examination.

**Fig. 1:**
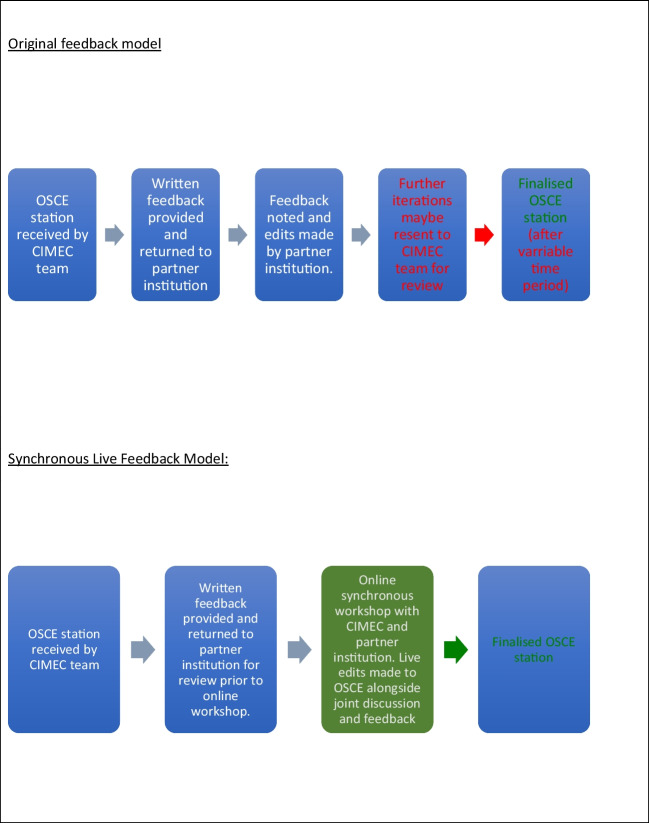
Original feedback model compared to live synchronous feedback model

A live online workshop was piloted providing a forum for discussion about the pre-submitted OSCE stations, which was attended by NGU and UCL faculty members (authors and reviewers). The live workshop utilised the model from previous collaborations and consisted of four subgroups in total (Fig. 1). Small groups with numbers limited to a maximum of six participants per group provided a platform for a two-way discussion about each OSCE station to be discussed. Groups remained consistent throughout the workshop to enhance rapport building. Faculty were assigned to groups based on their areas of expertise including their subspecialty.

Both teams prepared for the session by reviewing the OSCE stations and making notes where appropriate in advance. The workshop duration was 2 hours based on previous international workshops and feedback. A total of four OSCE stations were discussed in each subgroup where an open dialogue allowed an opportunity for opinions and debate to occur. This workshop model adopted a more collaborative approach and reduced the paternalistic elements of the previous feedback model adopted. Real-time edits were made on-screen by UCL facilitators with agreement from the NGU team. Feedback forms were disseminated after the workshop to the NGU faculty members to evaluate the workshop and its sustainability as a long-term adaptation to feedback delivery.

## Results

**Fig. 2:**
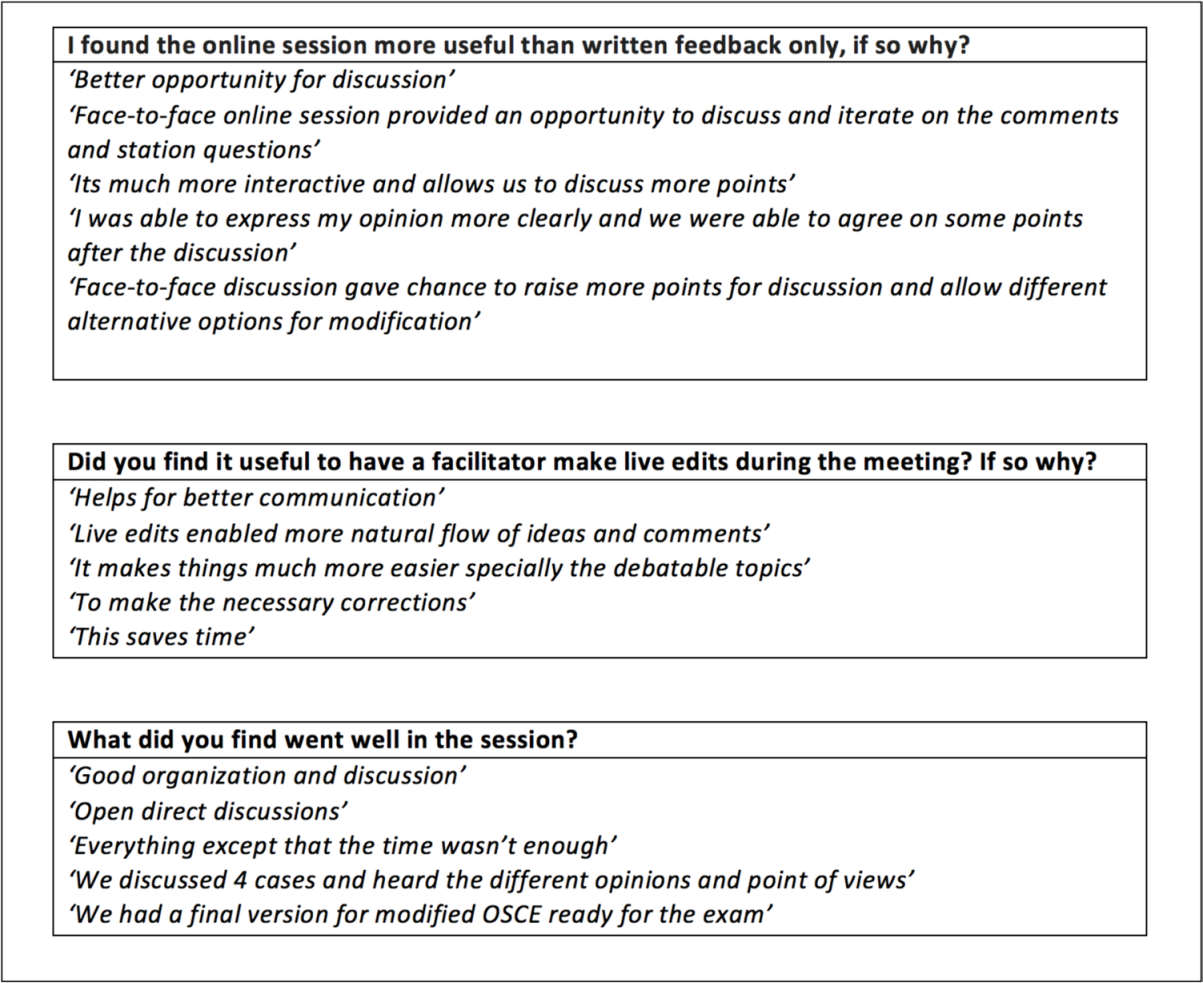
Evaluation feedback

Feedback was sought to evaluate the utility, efficiency and reception of the synchronous workshop in a mix of verbal and written formats and was resoundingly positive (Fig. 2). All participants identified the online session as more useful than written feedback alone, with 83% agreeing completely. All comments indicated that the synchronous workshop allowed for deeper discussion about OSCE format, timing within the station and logistics and practicalities of the station. Most participants also appreciated the process of live edits being made by the facilitator during the session as they felt that this better addressed their needs as a faculty and because it meant that a final version of the assessment was available by the end of the session. It also helped station authors to understand the rationale for, motivations behind, and context of, the reviewers’ feedback decisions. Future recommendations from NGU have included having more time allocated for discussion and a greater focus on exploring the challenges of developing OSCE stations and how these can be mitigated.

The findings of this feedback further reiterate that international educational partnerships are relational and greatly dependant on care and communication between partners, whereby the key to ensuring successful enduring partnerships lies in maintaining cross cultural relationships by employing humility, reflexivity and cognitive flexibility [10].

Moreover, our findings fit with the growing literature that feedback is a multifaceted phenomenon that is far more fluid in its concept than has traditionally been believed. Ajjawi et al. describe the concept of feedback as a co-constructive interaction that is inherently a fragile non-prescriptive process. Our experience has been consistent with this approach as feedback across partnerships often requires us to have difficult conversations in order to leverage change [11]. This can only be achieved where partnerships are strong and there is mutual respect and understanding. Our work also highlights that we must acknowledge the human elements of feedback literacy [12], which play a significant role in co-creation of educational and assessment material.

## Discussion

This co-designed OSCE feedback model, which harnesses the benefits of two-way dialogue, has led to an evolution in our joint approach to providing feedback for high-stakes assessment items in this ongoing partnership, especially for quality assuring OSCEs including fewer queries regarding stations.

We feel that this model may enable other partnering institutions to follow suit and will promote ongoing capacity building for both teams.

We hope to apply this synchronous feedback model to other assessments amongst our international partners when reviewing both written and practical assessments. Moreover, we believe that the scope of this collaborative model can also be further adapted to utilise within curriculum development review activities in the future.

## Data Availability

The data that support the findings of this study are available from the corresponding author AG upon reasonable request.
